# Hypoxic stress enhances extension and branching of dorsal root ganglion neuronal outgrowth

**DOI:** 10.1002/jsp2.1090

**Published:** 2020-05-04

**Authors:** Junxuan Ma, Despina Stefanoska, Laura S. Stone, Maria Hildebrand, Corrinus C. van Donkelaar, Xuenong Zou, Valentina Basoli, Sibylle Grad, Mauro Alini, Marianna Peroglio

**Affiliations:** ^1^ AO Research Institute Davos Davos Switzerland; ^2^ Department of Biomedical Engineering Eindhoven University of Technology Eindhoven The Netherlands; ^3^ Alan Edwards Centre for Research on Pain, Faculty of Dentistry McGill University Montreal, Quebec Canada; ^4^ Department of Spine Surgery, Orthopedic Research Institute The First Affiliated Hospital of Sun Yat‐sen University Guangzhou China; ^5^ Guangdong Provincial Key Laboratory of Orthopedics and Traumatology Guangzhou China

**Keywords:** cell viability, dorsal root ganglion, hypoxia, low back pain, neuronal outgrowth

## Abstract

It has been shown that painful intervertebral discs (IVDs) were associated with a deeper innervation. However, the effect of the disc's degenerative microenvironment on neuronal outgrowth remains largely unknown. The focus of this study was to determine the influence of hypoxia on dorsal root ganglion (DRG) neurite outgrowth. Toward this aim, the DRG‐derived cell line ND7/23 was either directly subjected to 2% or 20% oxygen conditions or exposed to conditioned medium (CM) collected from IVDs cultured under 2% or 20% oxygen. Viability and outgrowth analysis were performed following 3 days of exposure. Results obtained with the cell line were further validated on cultures of rabbit spinal DRG explants and dissociated DRG neurons. Results showed that hypoxia significantly increased neurite outgrowth length in ND7/23 cells, which was also validated in DRG explant and primary cell culture, although hypoxia conditioned IVD did not significantly increase ND7/23 neurite outgrowth. While hypoxia dramatically decreased the outgrowth frequency in explant cultures, it significantly increased collateral sprouting of dissociated neurons. Importantly, the hypoxia‐induced decrease of outgrowth frequency at the explant level was not due to inhibition of outgrowth branching but rather to neuronal necrosis. In summary, hypoxia in DRG promoted neurite sprouting, while neuronal necrosis may reduce the density of neuronal outgrowth at the tissue level. These findings may help to explain the deeper neo‐innervation found in the painful disc tissue.

**Highlights:**

Hypoxia promoted elongation and branching of neurite outgrowth at single cell level, but reduced outgrowth density at tissue level, possibly due to hypoxia‐induced neuronal necrosis; these findings may help to explain the deeper neo‐innervation found in clinically painful tissues.

## Graphical abstract

Deeper innervation has been observed in degenerative and painful intervertebral disc (IVD). Ischemia‐related deprivation of oxygen (hypoxia) is among the multiple factors that are associated with IVD degeneration and pain. The study investigated the influence of hypoxia in both peripheral nerve (namely dorsal root ganglion DRG) and IVD on aberrant neurite sprouting at both cell and tissue explant levels. Although hypoxia in IVD did not seem to produce an IVD conditioned medium that promoted neurite sprouting, hypoxia in DRG directly induced a longer neurite outgrowth with higher collateral sprouting. Additionally, hypoxia leaded to DRG neuronal necrosis, which might reduce the neurite frequency at the tissue explant level. Protecting DRG from hypoxic stress may be of importance in the treatment of discogenic pain.

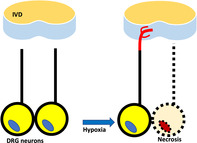

## 1 INTRODUCTION

Chronic low back pain (LBP) has been reported as the leading cause of disability worldwide, considering its incidence, prevalence, and years lived with disability.^1^ A major cause of chronic LBP is the degeneration of the intervertebral disc (IVD),^2^, ^3^ which is often associated with chronic inflammation, cell death, and extracellular matrix degradation.^4^, ^5^, ^6^ Deviations in nutrient supply of IVD are frequently associated with IVD degeneration.^7^, ^8^ Ischemia‐related risk factors such as aortic calcification, stenosis of lumbar arteries, smoking, and high serum cholesterol levels are consistently correlated with discogenic pain.^9^, ^10^ The association between permeability of the endplate and intervertebral disc degeneration has been described in human specimens since the seventies.^11^ Recently, endplate perfusion was evaluated in adult patients using dynamic contrast enhanced MRI and a reduced endplate perfusion was found to be correlated with a higher degree of IVD degeneration.^12^

One consequence of the decreased nutrient supply to the IVD is hypoxia, that is, an oxygen concentration below the metabolic requirements.^13^ An increased metabolic consumption of oxygen by resident IVD cells upon degeneration was recently reported;^14^ moreover, recruited inflammatory cells may further aggravate the hypoxic state.^15^ Hypoxia bio‐markers such as Hypoxia‐Inducible Factor 1‐alpha, Glucose Transporter Type 1, 3, and 9 were up‐regulated in clinical degenerative IVD samples compared to healthy controls.^16^, ^17^

While progress has been made to understand the mechanisms underlying disc degeneration, the most important clinical question concerns the pain related to disc degeneration.^18^ The healthy IVDs are almost aneural,^19^ but neo‐innervation has been repeatedly observed in painful and degenerative disc tissue^20^, ^21^ which represents a prospective vital factor for the mechanism of chronic pain. However, the pathophysiological mechanism of the aberrant nerve sprouting in the context of IVD hypoxia remains unknown.

The IVD exposed to hypoxia stress may therefore produce multiple factors stimulating the proximal sensory nerve structure named dorsal root ganglion (DRG), which is responsible for initiating nociceptive/pain input into the central nervous system for the sense of pain.^22^ This influence mediated via IVD (hypoxia stressed IVD may release molecules that stimulate DRG) was defined as the indirect effect of hypoxia on DRG.

On the other hand, the degenerative IVD can compress^23^ and chemically stimulate the DRG,^24^ increase its endoneural fluid pressure and reduce its blood supply,^25^ which is associated with the development of hypoxia in the DRG. Therefore, hypoxia may exert a direct influence on DRG. Although hypoxia in DRG has not been measured in discogenic pain patients so far, large animal models showed that exposure of nucleus pulposus tissue through IVD incision reduced blood flow in the nerve root and dorsal ganglion of dogs, therefore leading to DRG ischemia.^24^, ^26^ Furthermore, it has been shown that oxygen tension in DRGs was severely affected as a result of ischemia induced by aortic clamping.^27^

In a word, hypoxia was shown to play multiple roles in IVD degeneration and IVD‐related neuropathy in proximal DRG. The hypothesis of this study is that hypoxia may either directly or indirectly (via IVD) promote neurite sprouting which permits the neo‐innervation of IVD and can possibly be associated to chronic discogenic pain. Therefore, the aim of this study was to investigate (a) the effect of hypoxia‐stressed IVD conditioned medium (CM) on DRG neurite outgrowth and (b) whether hypoxia can directly promote neurite outgrowth in DRGs.

To study the indirect effect of hypoxia on DRG (via IVD), hypoxia was modeled by culturing whole IVDs from bovine tails inside an incubator set at 2% oxygen level. Since oxygen tension in the bone marrow above the IVD endplate was reported to be around 6.4%,^28^ 2% oxygen around the cultured IVD organ would meet the definition of hypoxia. Likewise, since normal oxygen tension in rat DRG tissue was estimated to be between 3.9% and 5.5%,^29^ 2% oxygen would also represent a hypoxic condition for DRG cell culture. The DRG‐derived ND7/23 cell line^30^, ^31^ was either cultured in hypoxia or in CM of hypoxia‐stressed IVD. Findings were validated using rabbit dissociated DRG neurons and adult rabbit DRG explant cultures.

## 2 MATERIALS AND METHODS

**FIGURE 1:**
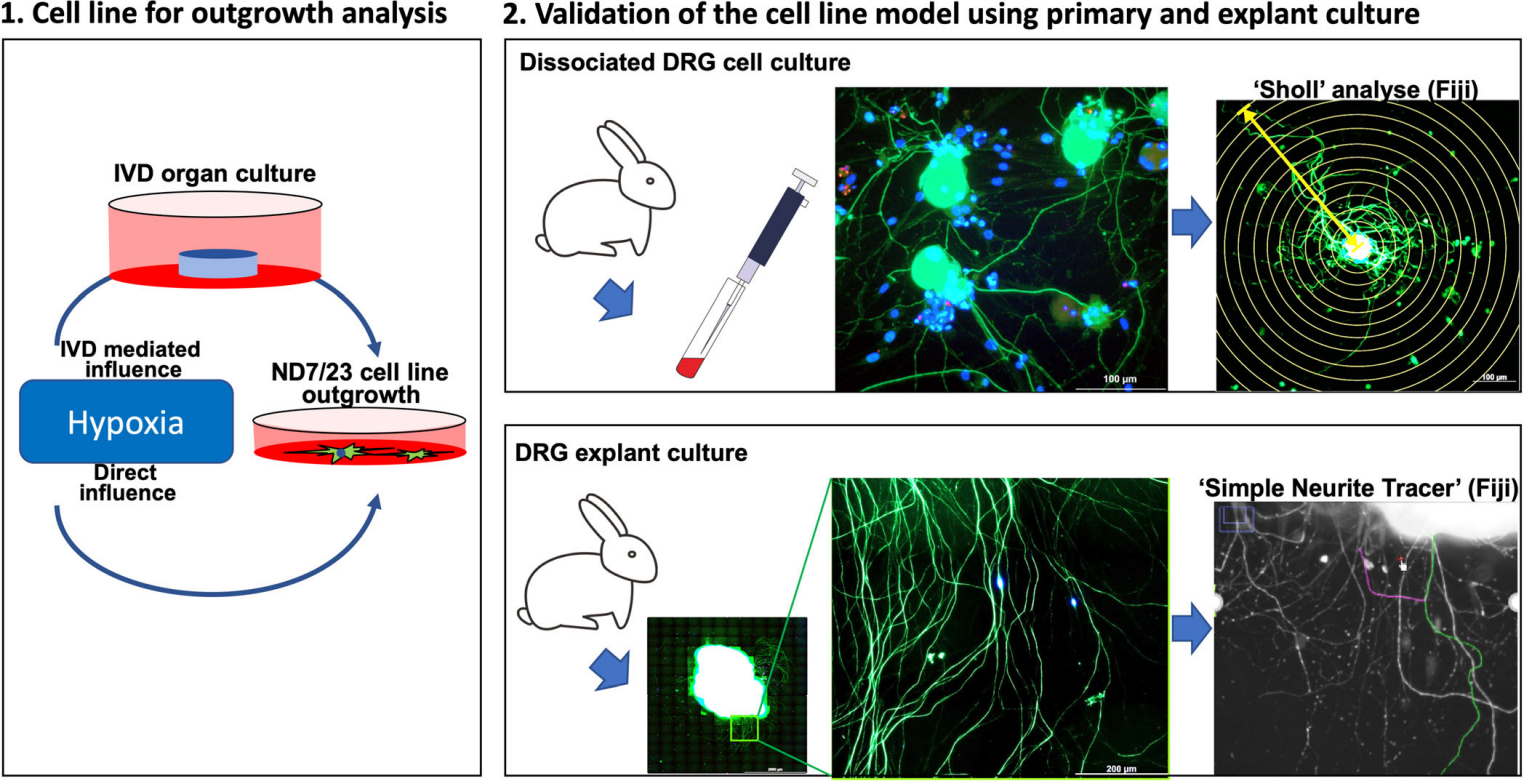
Experimental design. The direct and IVD mediated influence of hypoxia on DRG outgrowth was first evaluated using a cell line model and was validated by rabbit primary DRG culture and rabbit DRG explant culture

An overview of the experimental design used in this study is provided in Figure 1. Briefly, the effect of hypoxia on neurite outgrowth was initially tested using the DRG‐derived neuronal cell line ND7/23 and was then validated using primary DRG neurons or DRG explants that were obtained from the lumbar spines (L2‐5) of New Zealand white rabbits (n = 6, all female, 28 weeks old) from unrelated preclinical studies approved by the cantonal ethics committee of Graubünden/Grisons, Switzerland.

### 2.1 ND7/23 cell line culture and neural differentiation

DRG neuron‐derived ND7/23 cells (Sigma Aldrich, cat. n. 92090 903) were maintained in 4.5 g/L glucose Dulbecco's Modified Eagle Medium (DMEM, Gibco, cat. n. 52100‐021, UK), 10% fetal calf serum (FCS, Sera Plus, Biotech, cat. n. 3702‐P121812, DE), 1% penicillin/streptomycin (Gibco, cat. n. 15140‐122, UK), and 0.11 g/L sodium pyruvate (Sigma‐Aldrich, cat. n. P5280, JP). The cells were cultured at 37°C and 5% CO_2_ and passaged every 4 days.

Passage 8 cells were seeded at 10 000 cells/cm^2^ for the experimental setup. Since neural differentiation of the cell line can establish a long neurite outgrowth which is similar to that observed in primary sensory neurons in culture,^32^ neural differentiation was induced using 1 mM N6,2′‐*O*‐Dibutyryladenosine3′,5′‐cyclic monophosphate sodium salt (cAMP, Sigma‐Aldrich, D0260), 10 ng/mL recombinant rat beta‐nerve growth factor (NGF, R&D, 556‐NG‐100), 0.5% FCS in IVD CM or DMEM with 1% penicillin/streptomycin, and 0.11 g/L sodium pyruvate 1 hour after cell seeding and cell attachment.

### 2.2 The direct influence of hypoxia on neuronal outgrowth of the differentiated ND7/23 cell line

ND7/23 cells were seeded in 48‐well‐plates and were changed to neural differentiation medium 1 hour after cell seeding. Then the cells were cultured in either 2% oxygen or 20% oxygen by setting the oxygen level of the incubator. After 72 hours, 10 random fields of each well were captured by phase contrast at ×20 magnification, 0.40 numerical aperture and 6.8 mm working distance using EVOS FL Auto 2 Imaging System (Thermo Fisher Scientific). The duration of DRG cell line treatment was limited to 72 hours because a longer culture time in low serum conditions caused high cell death.

The longest neurite of each cell was measured using the “simple neurite tracer” plugin (version: 3.1.3) within ImageJ Fiji (version: 1.52p, NIH).^33^ To exclude cell extensions or elongations from outgrowth analysis, only neurites longer than 30 μm were defined as neuronal outgrowth. The proportion of cells with outgrowth per field was referred to as outgrowth frequency.^34^

### 2.3 The influence of hypoxia‐stressed IVD CM on neuronal outgrowth of the differentiated ND7/23 cell line

IVDs were dissected from seven bovine tails obtained from a local abattoir. Two whole IVD organs with intact endplates were excised from each tail and cultured under free‐swelling conditions according to former reports^35^, ^36^ for 3 days in either 2% or 20% oxygen. The IVD culture medium was DMEM supplemented with 10% fetal calf serum, 1% penicillin/streptomycin and 0.11 g/L sodium pyruvate. The donor and IVD size were equally distributed between groups. Following this treatment, all the discs were cultured in DMEM supplemented with 20 mM HEPES (Thermo Fisher, cat. n. 15630122) under 20% O_2_ for another 24 hours. The latter CM was collected and stored at −20°C until further analysis.

ND7/23 cells were seeded in 48‐well‐plates and treated with the different IVD CM. Additionally, 0.5% FCS, 1 mM cAMP, and 10 ng/mL (NGF) were added to all the groups to induce neural differentiation. After 72 hours of culture, 10 random fields of each well were captured by phase contrast at ×20 magnification, 0.40 numerical aperture, and 6.8 mm working distance using EVOS FL Auto 2 Imaging System. The outgrowth evaluation method was the same as described above.

### 2.4 Immunofluorescence of HIF‐1 alpha and viability of the differentiated ND7/23 cell line at 2% and 20% oxygen

To evaluate cell viability and confirm hypoxia in the cells, the differentiated ND7/23 cells were cultured in hypoxia/ normoxia for 72 hours in poly‐l‐lysine coated 15 μ‐Slide eight‐well chambers (80 824, ibidi, DE). Cells were then incubated with 30 μM ethidium homodimer‐1 for 15 minutes at 37°C and then fixed in 4% buffered formalin for 30 minutes at room temperature (Formafix, cat. n. 1803 032, CH). Following three washes with deionized water, immunofluorescent staining using HIF‐1 alpha antibody (1:500 incubation at 4°C overnight, Novus Biologicals, cat. n. NB100‐479) with Alexa Fluor Plus 680 conjugated secondary antibody (1:500 incubation at room temperature for 1 hour, Thermo Fisher, cat. n. A32802) and Hoechst nuclei staining (1 μM, Sigma, cat. n. 14530) was performed. Stained cells were imaged at an excitation wavelength of 628, 531, and 357 nm for HIF‐1 alpha label, ethidium homodimer‐1, and Hoechst staining, respectively, using EVOS FL Auto 2 Imaging System at ×20 magnification, 0.40 numerical aperture, and 6.8 mm working distance. Necrotic neurons were identified by the nucleus staining of ethidium homodimer‐1, while neuronal apoptosis was assessed by nucleus morphology of karyopyknosis or karyorrhexis. The cell density, the proportions of necrotic, apoptotic and viable cells, and HIF‐1 alpha staining fluorescent intensity normalized by cell number were then calculated in each field. Three independent experiments were performed at different times using different vials of thawed cells, and each independent experiment included 5 to 7 wells of culture per group.

### 2.5 RNA isolation and real‐time PCR on differentiated ND7/23 cells

The differentiated ND7/23 cells were cultured in hypoxia/ normoxia for 72 hours in 25 cm^2^ culture flasks (hypoxia and normoxia each included 2 flasks as technical replicates). At the end of culture, cells were lysed in 1 mL of TRI Reagent (TR 118, MRC, OH). Total RNA was extracted according to manufacturer's instructions. RNA concentration and purity were determined by Nanodrop 1000 Spectrophotometer (ThermoFisher, Waltham, MA). The gene expression assays were all purchased from Applied Biosystems (ThermoFisher). The mRNA expression of von Hippel‐Lindau tumor suppressor (rat Vhl, Rn00583795_m1), B‐cell CLL/lymphoma 2 (rat Bcl2, Rn99999125_m1), BCL2‐associated agonist of cell death (rat Bad, Rn00575519_m1), Bcl2‐associated X protein (rat Bax, Rn01480161_g1), and hypoxia‐inducible factor 1, alpha subunit (rat Hif1a, Rn01472827_g1) genes were quantified by Real‐time PCR analysis. For cDNA preparation, 1 μg of RNA (A260/A280 > 1.8) was reverse‐transcribed by TaqMan Reverse Transcription Reagents (×10 PCR Buffer II, 25 mM Magnesium chloride, DeoxyNTPs mixture, Random Hexamer, RNase Inhibitor, and MultiScribe Reverse Transcriptase [50 U/μl]; all from Applied Biosystems, CA) according to manufacturer's instructions. PCR amplification was performed in 10 μL reaction volume, using TaqMan Universal Master Mix (cat. n. 4304437, ThermoFisher, CA), in a Step One Plus Real‐Time PCR System (Applied Biosystems, Foster City, CA). Gene expression relative to 20% oxygen control was assessed by the 2^‐ΔΔ*Ct*^ method. Eukaryotic 18S rRNA was used as endogenous control (18S, Hs99999901_s1) (cat. n. 4333760 T).

### 2.6 Neuronal outgrowth of DRG explants at 2% and 20% oxygen

The DRG explant culture was performed based on the protocol described by Buyens et al, with minor modifications.^37^ Briefly, DRGs were harvested from the lumbar spines (L2‐5) of four New Zealand white rabbits (female, 28 weeks old) obtained from unrelated preclinical studies approved by the cantonal ethics committee of Graubünden / Grisons. After carefully removing the nerve roots and membrane, DRGs were cut in half along their axis and seeded onto coverslips (25 × 25 mm^2^) (Menzel Gläser, DE) coated with 100 μg/mL poly‐d‐lysine (cat. n. P6407) for 1 hour at room temperature and followed by 2 μg/mL Laminin (cat. n. L2020) incubation at 37°C overnight (both from Sigma‐Aldrich). The culturing medium was DMEM/F12 (50% v/v, DMEM from Gibco, 52100‐021, UK and F‐12 Ham from Sigma, N6760, UK) supplemented with 10% fetal calf serum (Sera Plus, Biotech, 3702‐P121812, DE), 1% penicillin/streptomycin (Gibco, 15140‐122, UK), and 0.11 g/L sodium pyruvate (Sigma‐Aldrich, P5280, JP). DRGs from the same segment were assigned to 2% and 20% oxygen and cultured in an incubator at 5% CO_2_ and 37°C for 4 days. A 4‐day culture was chosen since we previously observed that DRG explants exhibit maximum outgrowths at 4 days when cultured without exogenous growth factors. Afterwards, DRGs were fixed in 4% buffered formalin (Formafix, cat. n. 1803032, CH) at room temperature for 30 minutes and washed with deionized water for 3 times. The neuronal outgrowth was immunostained by the anti‐neurofilament mouse monoclonal antibody (NF‐200, 1:100 incubation at 4°C overnight) (Thermo scientific, cat. n. OMA1‐06117, The Netherlands) diluted in PBS containing 0.1% Triton‐X (Sigma, cat. n. T8787) and 0.5% goat serum (1:20, vector laboratories, cat. n. S‐1000) after blocking with 5% goat serum blocking solution at room temperature for at least 2 hours. A polyclonal goat anti‐mouse AlexaFluor 488 conjugated antibody (1:100 incubation at room temperature for 1 hour, Thermo Fisher, cat. n. A‐11029) was used as the secondary antibody. For all the immunofluorescent stainings, omitting the primary antibody served as a negative control. Images of the whole DRG with the outgrowth were acquired using EVOS FL Auto 2 Imaging System at an excitation wavelength of 445 nm, ×20 magnification, 0.40 numerical aperture, and 6.8 mm working distance. The total frequency and length of neurite outgrowth was measured by the “simple neurite tracer” plugin (version: 3.1.3) within ImageJ Fiji (version: 1.52p, NIH)^33^ and averaged per explant.

### 2.7 Viability of DRG‐isolated neurons at 2% and 20% oxygen

The DRG cell dissociation and culture were modified based on the previous report.^38^ DRGs were dissected from two rabbit lumbar spines (L2‐5) (New Zealand white female rabbits, 28 weeks old). The isolated cells were obtained by digestion of the half‐cut DRGs with 2.5 mg/mL type I collagenase (Sigma‐Aldrich, cat. n. C9896) in phosphate‐buffered saline (37°C, on a shaker) for 1 hour and trituration of the loosened DRGs with a fine pipette tip (10‐200 μL) in 2 mL of 0.25% trypsin/EDTA (Gibco, cat. n. 15400‐054, UK) solution (prewarmed to 37°C) by pipetting 10 times. The supernatant was collected in DMEM/F12 medium with 10% FCS after the undissociated material sedimented. To improve cell yield, 2 mL of trypsin/EDTA were added to the undissociated material and the cell collection procedure was repeated twice. The collected cells were filtered using a 100 μm cell strainer (Falcon, Corning) and were resuspended in DMEM/F12 medium with 10% FCS. 50 μL of cell suspension containing around 5000 cells were plated at the center of each PDL/Laminin coated coverslip (25 × 25 mm^2^). Following an overnight incubation, the cultures were supplied with another 1 mL of DMEM/F12 medium with 10% of FCS and were further maintained in 2% or 20% oxygen for 4 days.

The viability and outgrowth of the DRG‐isolated neuron culture were evaluated based on the protocol described by Gladman et al.^39^ Briefly, DRG isolated cells were incubated with 3 μM ethidium homodimer‐1 for 15 minutes at 37°C, and then fixed in 4% buffered formalin at room temperature for 30 minutes (Formafix, cat. n. 1803032, CH). Following three washes with deionized water, immunofluorescent staining using NF200 (1:100, Thermo scientific, cat. n. OMA1‐06117, The Netherlands) with AlexaFluor 488 conjugated secondary antibody (1:100, Thermo Fisher, cat. n. A‐11029) and Hoechst nuclei staining were performed. Stained cells were imaged at an excitation wavelength of 470, 531, and 357 nm for NF200 label, ethidium homodimer‐1, and Hoechst staining, respectively, using EVOS FL Auto 2 Imaging System. Necrotic neurons were identified by the co‐staining of NF200 and ethidium homodimer‐1, while neuronal apoptosis was assessed by nucleus morphology of karyopyknosis or karyorrhexis of NF200 positive neurons. The proportions of neuronal necrosis and apoptosis among all neurons in each field were then calculated.

### 2.8 Neuronal outgrowth of DRG‐isolated neurons at 2% and 20% oxygen

Since most outgrowths of nearby neurons intersected with each other at 4 days, a 2‐day culture of primary neurons of rabbit DRG was performed to evaluate outgrowth at a single cell level (obtained from L2‐5 of 2 New Zealand white female rabbits, 28 weeks old). The methods for cell culture, immunofluorescent labelling, ethidium homodimer‐1, and Hoechst staining were identical to the ones used for the 4‐day primary neuron culture. Images were acquired with EVOS FL Auto 2 Imaging System at ×20 magnification, 0.40 numerical aperture, and 6.8 mm working distance to visualize the neuronal soma and its outgrowth.

The selection of neurons for the evaluation of outgrowth was based on the following criteria: (a) the cell was identified as neuron by positive staining to NF200; (b) no sign of necrosis or apoptosis was detected; and (c) the outgrowths of the neurons were not contacting or intersecting with one another. The mask of the neuronal outgrowth was created using ImageJ with a threshold of 35‐255. The “particle remover” plugin of ImageJ Fiji was used to eliminate the background. The neurons were analyzed using the “Sholl” method for the length, density, and pattern distribution of outgrowth.^40^ Three regions (<30, 40‐105, and 120‐150 μm from soma) were defined, and categorized as short, intermediate, and far regions from the soma. Outgrowths from the three areas were separately analyzed for the distribution of collateral sprouting.

### 2.9 Statistics

For the cell line, DRG explant culture and isolated primary neuron culture, data acquired for the outgrowth and viability were not normally distributed, so the median values of the 2% and 20% oxygen culture groups were compared using a Mann‐Whitney test. *P* values lower than .05 were considered significant. Statistical analyses were performed using SPSS 16.0 (SPSS Inc.).

For the outgrowth of ND7/23 cells in IVD CM, CM from seven bovine tails were separately applied to stimulate the ND7/23 cells. IVD CM from each tail was used to stimulate three wells of ND7/23 culture, and one field per well was taken for outgrowth evaluation. The results shown are the combined data from all the seven tails. For the outgrowth and viability of ND7/23 cells in 2% and 20% oxygen, the data shown are pooled from six independent experiments performed with two or three wells per condition and imaging of one random field per well.

For the viability and immunofluorescence of ND7/23 cells in 2% and 20% oxygen, three independent repeats were performed. Each independent experiment included 6 to 8 wells of culture, one of the wells was used as negative control of immunofluorescence without primary antibody. Five to ten fields of each well were taken to evaluate HIF‐1α expression, thus 35 to 70 fields were used for the evaluation. The data shown are taken from one representative experiment as all three independent experiments showed the same trend. For RT‐PCR analysis, two independent replicates were performed, each independent replicate (performed at different time) had two technical replicates (performed at the same time but from different flasks of culture).

For the explant cultures, 48 DRGs halves (from 4 rabbits, 3 spine levels/rabbit, 2 DRGs/spine level, each DRG cut in half) were collected (24 samples/group). DRG halves detached during culture were excluded from the analyses (2‐4 samples/group).

For the DRG dissociated cells cultured for 4 days to evaluate viability, experiments were conducted with cells from two different rabbits and two wells/donor and condition were analyzed. A total of 100‐134 fields/well were assessed (210 fields for 2% oxygen and 223 fields for 20% oxygen). Both donors showed similar trends for apoptosis and necrosis at both oxygen levels.

For the DRG dissociated cells cultured for 2 days to evaluate outgrowth pattern, experiments were conducted with cells from two different rabbits and two wells/donor and condition were analyzed. Only neurons that did not have intersections with other neurons (25 neurons in 2% oxygen and 27 neurons in 20% oxygen) were analyzed using the “Sholl” method.

## 3 RESULTS

### 3.1 Hypoxia induced longer neurite outgrowth in ND7/23 cell line

**FIGURE 2:**
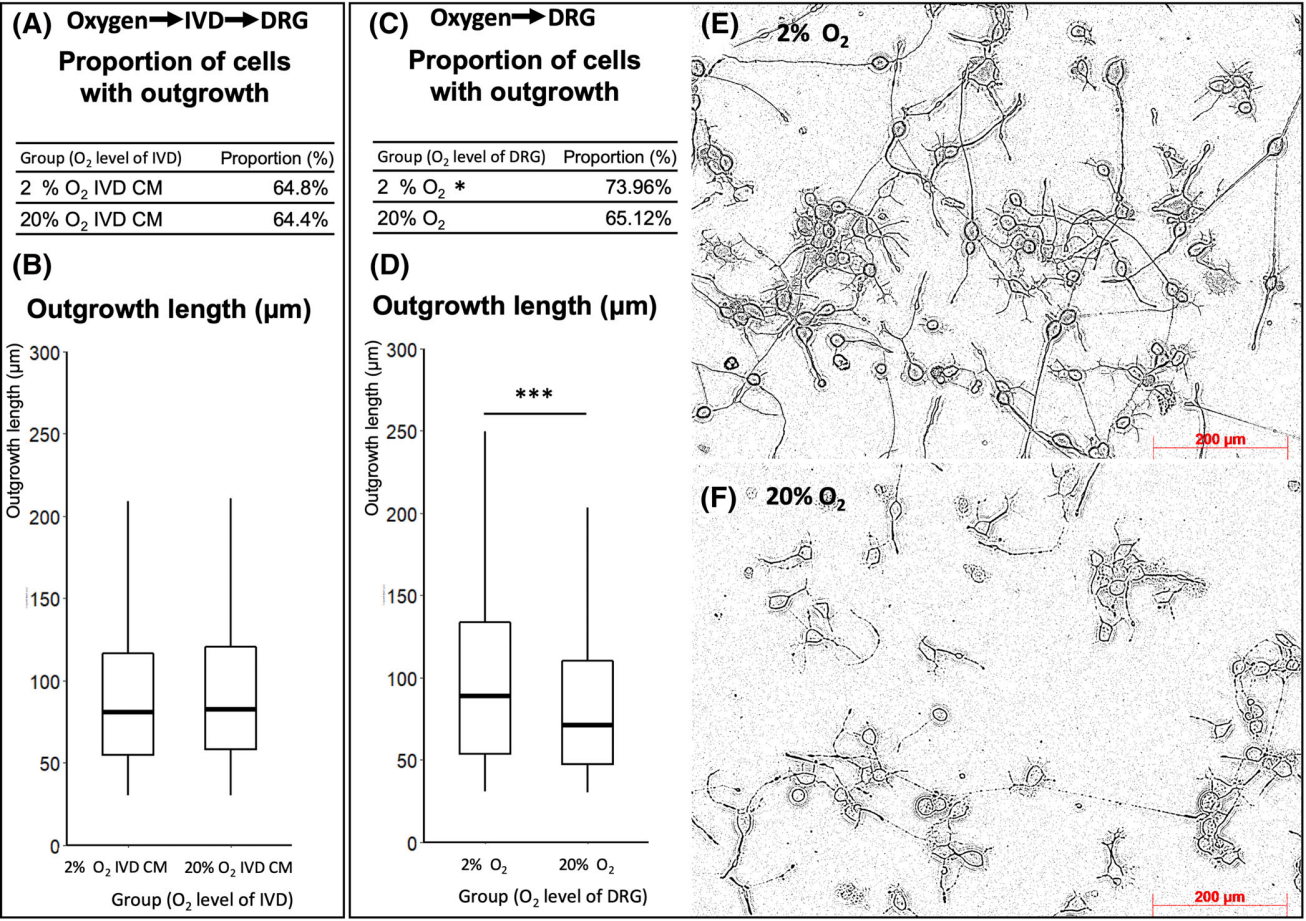
Neuronal outgrowth evaluation of ND7/23 cell line after culturing at 2% and 20% oxygen for 3 days. Phase contrast images were taken and analyzed using NeuronJ plugin of ImageJ Fiji. A,B, The proportion of neurite outgrowth and the length of outgrowth stimulated by the IVD CM was not significantly influenced by the oxygen level of IVD culture. (Chi‐square test for proportion of cells with outgrowth, Mann‐Whitney test for outgrowth length between groups, *P < .05, n = 571 and 500 cells for 2% oxygen IVD CM and 20% oxygen IVD CM). C, D The proportion of neurite outgrowth and the length of outgrowth were all significantly higher for 2% than 20% oxygen at 3 days (Chi‐square test for proportion of cells with outgrowth, Mann‐Whitney test for outgrowth length between groups, *P < .05, ***P < .001, n = 265 and 410 cells for 2% and 20% oxygen). For B,D, a box plot was used to represent the data since it was not normally distributed. The central rectangle spans the interquartile range; the segment inside the rectangle shows the median; and whiskers above and below show the minima and maxima. E,F, Representative phase contrast images of ND7/23 cells cultured at 2% and 20% oxygen for 3 days. Outgrowth examples are indicated by yellow arrows. Scale bars equal 200 μm

Since DRGs may be directly influenced by hypoxic stress, the influence of hypoxia on neurite outgrowth was evaluated. Results showed that hypoxic culture conditions increased the proportion of cells with outgrowth by 13.6% (*P* = .02; Figure 2C) and increased the median outgrowth length by 38.5% (*P* < .001) (Figure 2D) at 3 days compared to normoxic cultures. Representative phase contrast images of the cells cultured at 2% and 20% oxygen are provided in Figure 2E,F.

### 3.2 Hypoxia on IVD was not found to influence outgrowth of ND7/23 cell line

When exposed to hypoxia, factors produced by the IVD were hypothesized to influence DRG neurite outgrowth. The results, however, did not seem to support this hypothesis. The IVDs treated with hypoxia showed no significant difference comparing to those treated by normoxia regarding the influence of their CM on neurite outgrowth of ND7/23. (Figure 2A,B).

### 3.3 Hypoxia increased *Vhl* gene expression and HIF‐1α protein level in the DRG cell line

**FIGURE 3:**
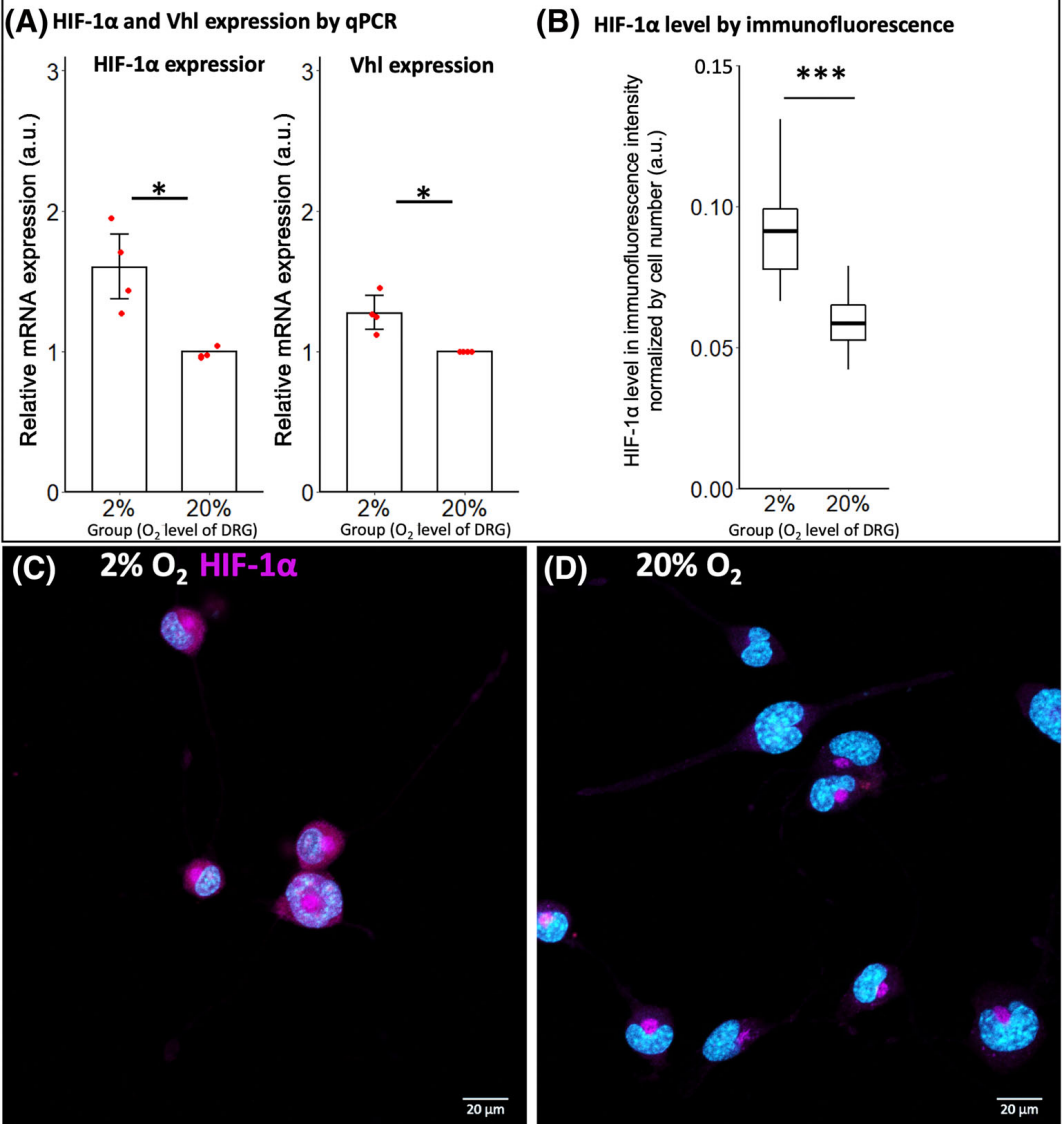
Hypoxia biomarker expression of ND7/23 cells after culturing at 2% and 20% oxygen for 3 days. A, Quantitative RT‐PCR showed that 2% oxygen significantly increased the relative mRNA expression of *Hif*‐1α and *Vhl* than 2% oxygen (*P < .05 by Mann‐Whitney test, n = 4 technical replicates for both 2% and 20% oxygen from two independent experiments). B, Immunofluorescence showed that the protein level of Hif‐1α was significantly higher for 2% oxygen than for 20% oxygen (*P < .05 by Mann‐Whitney test, n = 40 fields for both 2% and 20% oxygen). C,D, Representative image of ND7/23 cells immune‐stained by Hif‐1α antibody. Higher fluorescent staining was observed in soma of cells cultured in 2% oxygen than 20% oxygen

To identify if hypoxia was successfully induced in the DRG cell line using 2% oxygen culture, a biomarker of hypoxia was evaluated at both mRNA and protein level using quantitative RT‐PCR and immunofluorescence evaluation. Results showed the upregulation of the expression of *Hif*‐1α and *Vhl* genes, which are well established markers for the cellular response to hypoxia,^41^, ^42^ when the DRG cell line was cultured under 2% oxygen for 3 days (Figure 3A). The increased expression of Hif‐1α was also confirmed at the protein level by immunofluorescence (Figure 3B). Representative immunofluorescent images are shown in Figure 3C,D.

### 3.4 Hypoxia decreased DRG cell line viability

**FIGURE 4:**
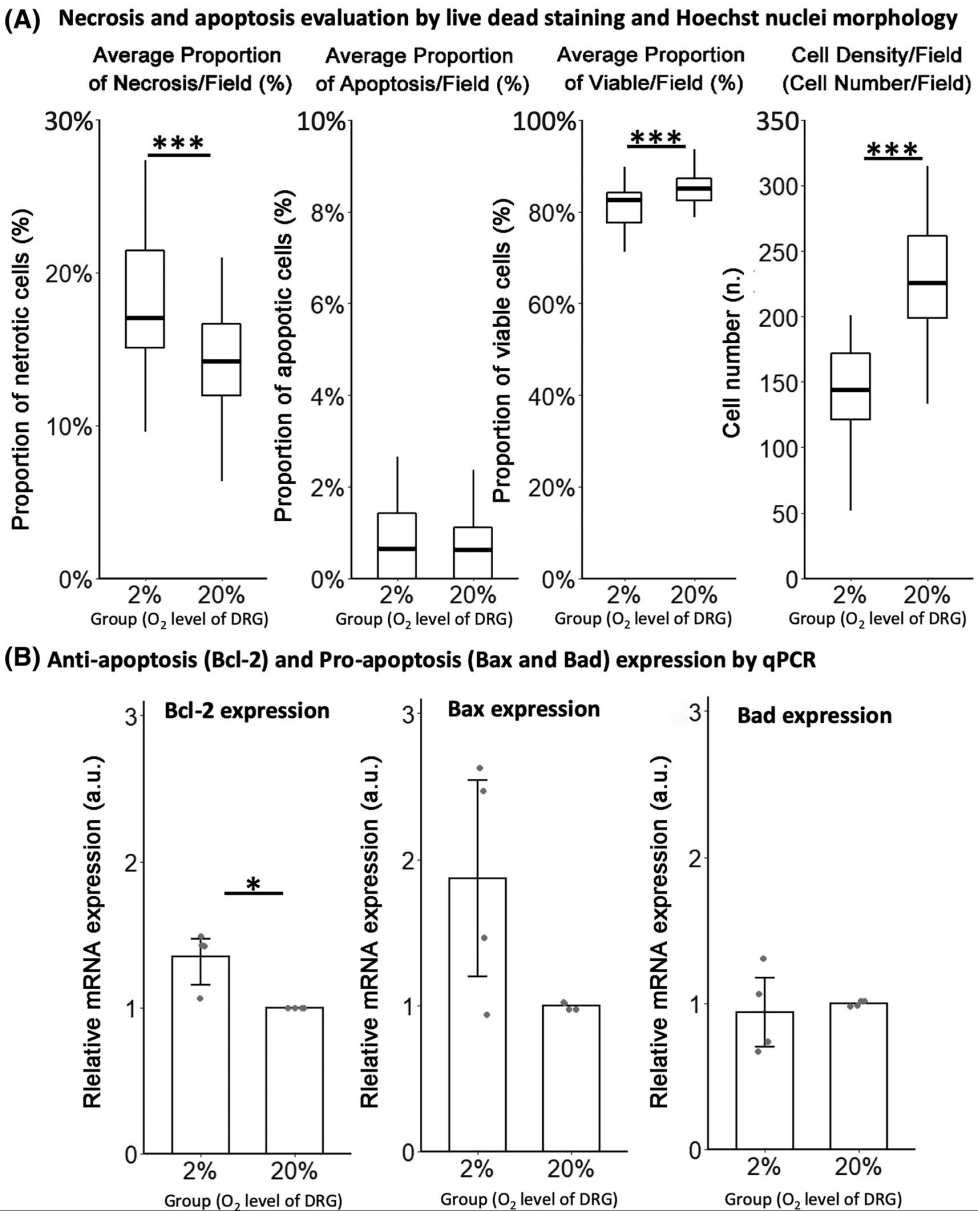
Viability of differentiated ND7/23 cells following a 3‐day culture at 2% and 20% oxygen. A, Hypoxia induced a significantly higher proportion of necrotic cells, a significantly lower proportion of viable cells and a reduced cell density compared to culture at 20% oxygen (*P < .05 by Mann‐Whitney test, n = 40 fields for both 2% and 20% of oxygen). B, 2% oxygen significantly increased the relative mRNA expression of the anti‐apoptotic*Bcl‐2* gene, while the pro‐apoptosis genes *Bax* and *Bad* showed no significant difference. The *Bad*/*Bcl‐2* was even significantly lower under 2% oxygen (*P < .05 by Mann‐Whitney test, n = 4 technical replicates for both 2% and 20% oxygen from 2 independent experiments). Data were normalized to normoxia control

Viability was evaluated in a separate 2‐day experiment using ethidium homodimer‐1 staining (necrosis) and nuclei morphology (apoptosis) analysis. mRNA expression of anti‐apoptosis (*Bcl‐2*) and pro‐apoptosis (*Bax* and *Bad*) genes^43^ was evaluated using quantitative RT‐PCR. Results showed that hypoxia decreased the median proportion of viable cells by 3.0% (*P* < .001) without considering the detached dead cells, and reduced cell density by 84.7% (*P* < .001) (Figure 4A).

Decreasing oxygen tension increased the median proportion of necrotic cells per field by 19.2% (*P* < .001) (Figure 4A). Although by nuclei morphology analysis, the proportion of apoptotic cells per field was not significantly influenced by hypoxia (*P* = 1) (Figure 4A), quantitative RT‐PCR showed a significantly increase in anti‐apoptosis gene expression in the hypoxic group (*P* < .001) (*Bcl‐2*), while pro‐apoptosis genes (*Bax* and *Bad*) were not significantly different between the culture groups (for *Bax*, *P* = .28) (for *Bad*, *P* = 1) (Figure 4B). These results indicate the reduced viability in hypoxia is mainly due to higher necrosis.

### 3.5 Hypoxia increased length but decreased frequency of outgrowth of 4‐day‐cultured DRG explants

**FIGURE 5:**
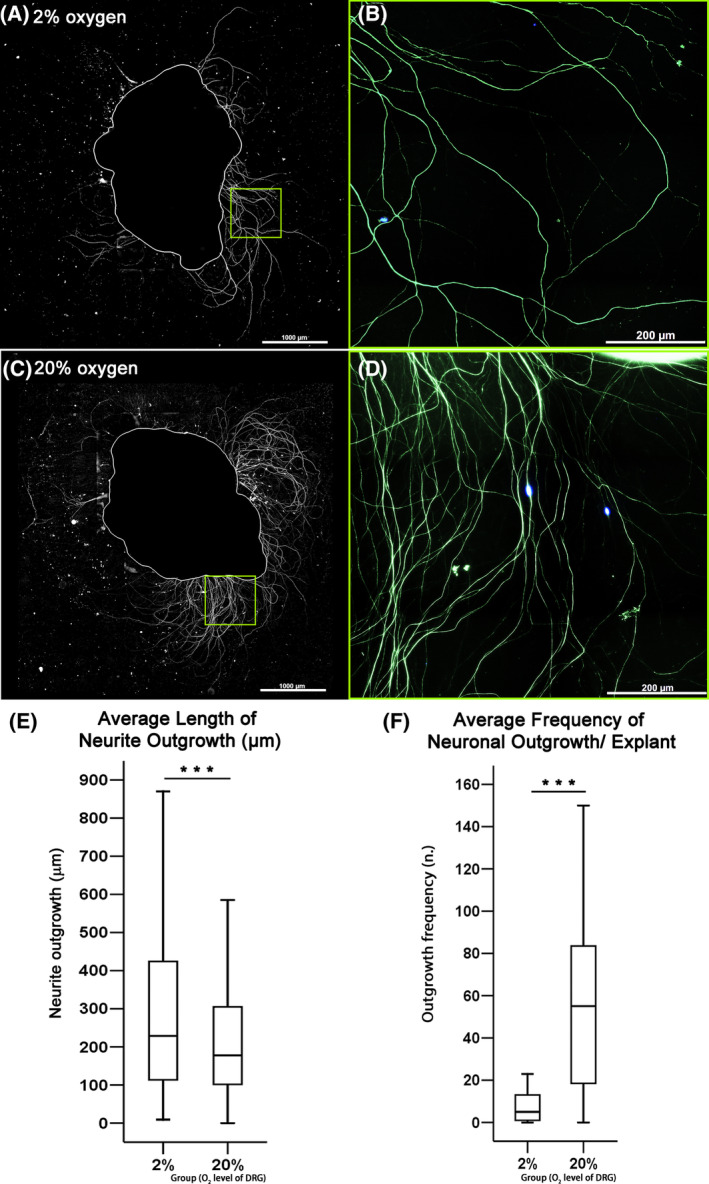
Evaluation of neuronal outgrowth based on the 4‐day culture of DRG explants obtained from rabbit lumbar spines. Immunofluorescent staining with neurofilament NF‐200 antibody was used to identify neuronal outgrowth. Data from four rabbit donors were pooled for quantification. A,C, Representative images of outgrowth from DRG explant at 2% or 20% oxygen (scale bars equal 1000 μm). B,D, Magnified view of the regions of interest in A,C showing that the density of outgrowth at 20% oxygen was higher than at 2% oxygen (scale bars equal 200 μm). E, Image analysis showed that 2% oxygen induced significantly longer neuronal outgrowth than 20% oxygen (***P < .001 by Mann‐Whitney test, n = 230 and 1131 nerve fibers for 2% and 20% oxygen respectively). F, Frequency of neuronal outgrowth per DRG was significantly larger for 20% than for 2% oxygen (***P < .001 by Mann‐Whitney test, n = 22 and 20 explants for 2% and 20% oxygen, respectively)

DRG explant culture was used to verify the findings obtained with the cell line. To attain equal cultured DRG size between 2% and 20% oxygen, at each spinal level, DRG halves from one side were assigned to 2% oxygen, while halves from the other side were cultured under 20% oxygen (the assignment of the side was performed randomly). After 4 days of culture, NF200 stained outgrowth density was lower under 2% oxygen as shown in Figure 5A‐D. Removal of primary antibody resulted in the absence of positive staining (data not shown). Quantification using the Simple Neurite Tracer plugin within ImageJ showed that outgrowth length was 28.6% higher under 2% oxygen (*P* < .001) (Figure 5E), but total neurite outgrowth frequency per DRG was reduced by 90.9% at 2% as compared to 20% oxygen (*P* < .001) (Figure 5F).

### 3.6 Hypoxia led to a higher neuronal necrosis of 4‐day‐cultured DRG primary neurons

**FIGURE 6:**
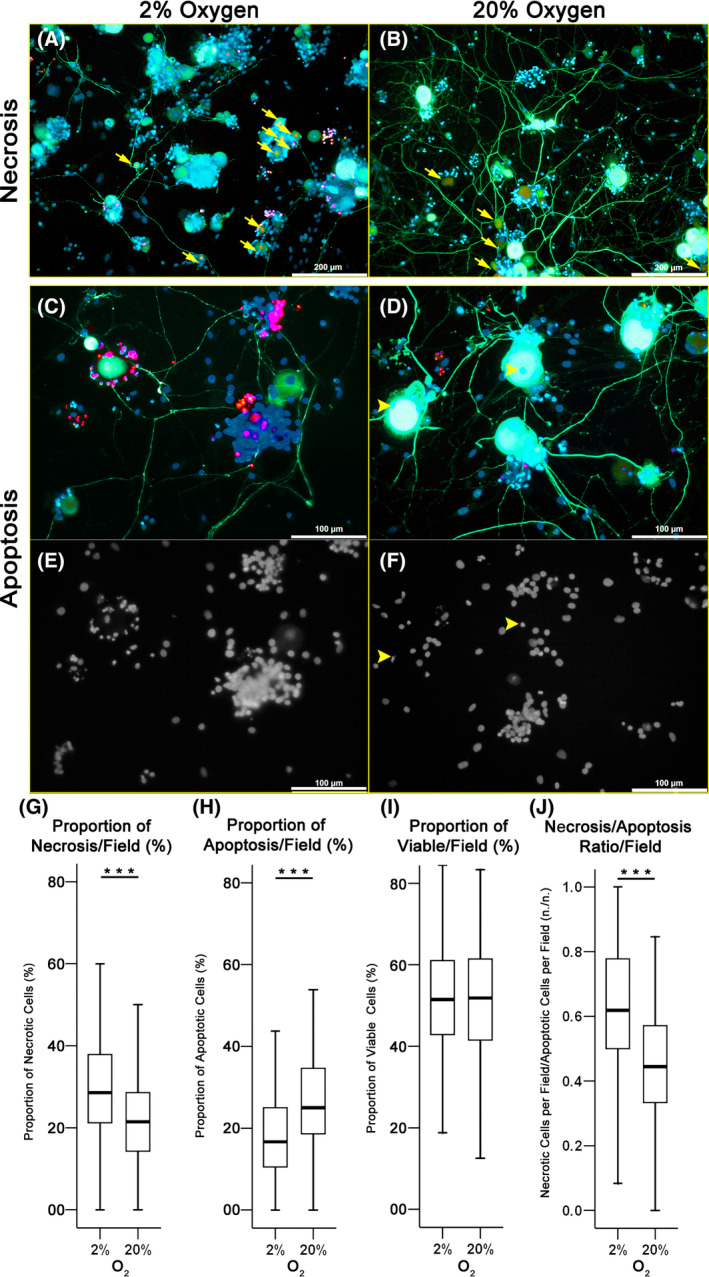
Viability of a 4‐day primary culture of isolated DRG neurons. The neurons were isolated from the DRGs of two rabbit spines. A,B, Representative images of DRG cells cultured at 2% and 20% oxygen for 4 days. Immunofluorescent staining with NF‐200 antibody was used to distinguish neurons from other cell types and ethidium homodimer‐1 was used to detect necrosis, which is shown by the arrows in the image. Scale bars equal 200 μm. C‐F, When combining Hoechst staining with immunofluorescence, neuronal apoptosis could be observed as indicated by arrow heads. Scale bars equal 100 μm. G, Under 2% oxygen a significantly higher proportion of necrotic cells in each field was observed than in cultures kept at 20% oxygen. H Significantly lower proportion of apoptotic cells per field was found at 2% oxygen. I, No significant difference was identified for the proportion of viable neurons at 2% and 20% oxygen (*P* = .769). J, The necrosis/apoptosis ratio per field was significantly higher for 2% than for 20% oxygen (***P < .001 by Mann‐Whitney test, n = 210 and 223 fields for 2% and 20% oxygen, respectively)

To investigate whether the reduced frequency of outgrowth for the DRG could be attributed to neuronal necrosis, primary DRG neurons were cultured for 4 days under 2% and 20% oxygen and cell viability was evaluated. Neurons were identified using NF200 immunostaining and removal of primary antibody resulted in the absence of positive staining. Indeed, the proportion of neuronal necrosis per field was increased by 33.3% at 2% compared to 20% oxygen (*P* < .001) (Figure 6A,B,G). The proportion of apoptotic cells in 2% oxygen was 49.5% lower than in 20% oxygen (*P* < .001) (Figure 6C‐F,F). Overall, the proportion of viable neurons (non‐necrotic and non‐apoptotic) was similar between 2% and 20% oxygen (0.8% lower for 2% oxygen, *P* = .77) (Figure 6I), but the necrosis/apoptosis ratio was significantly higher for the culture at 2% oxygen (39.3% higher, *P* < .001; Figure 6J).

### 3.7 Hypoxia led to longer neurites with higher branching in cultured DRG primary neurons

**FIGURE 7:**
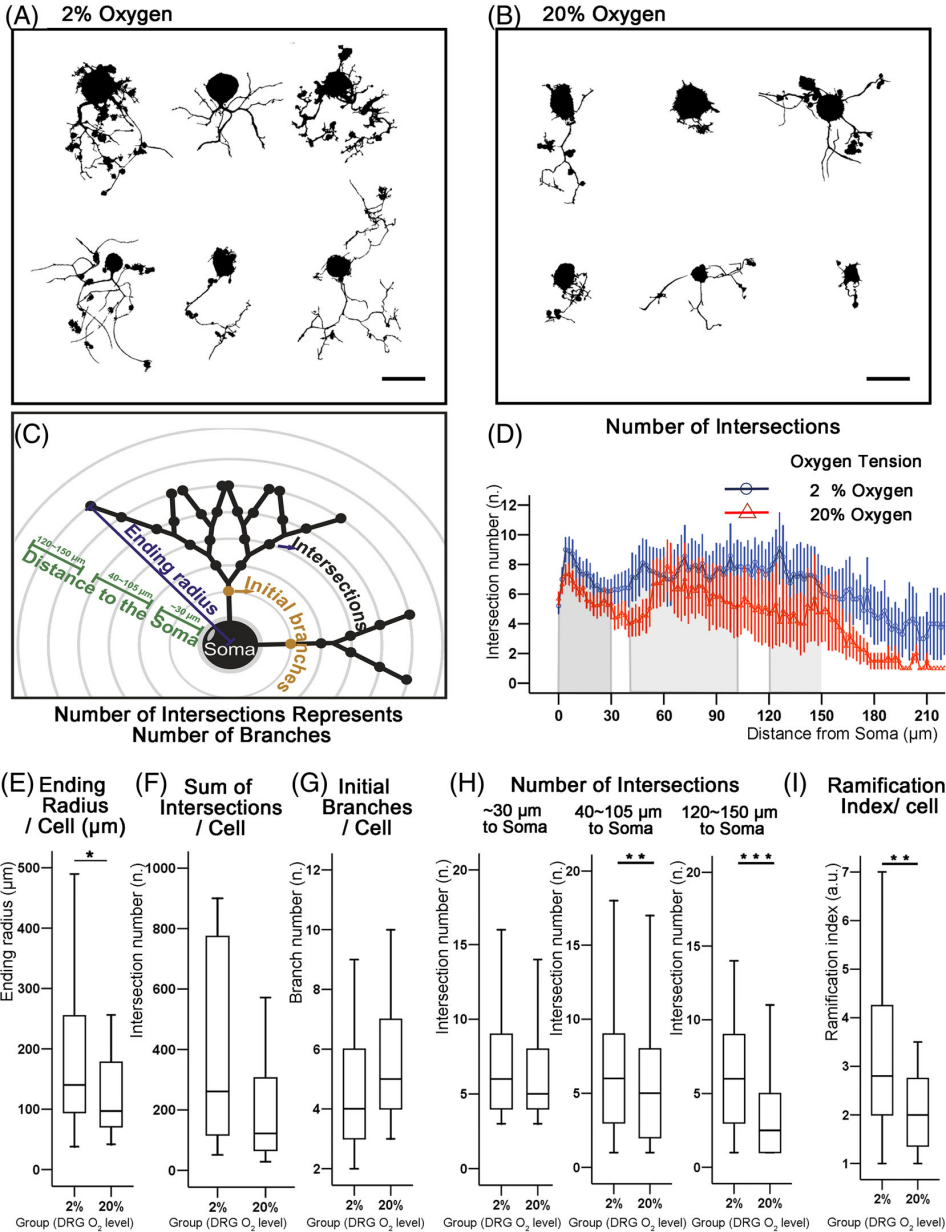
“Sholl” analysis of DRG neurons cultured at 2% and 20% oxygen for 2 days. A,B, Pattern images of DRG neurons (immunofluorescent staining with NF‐200 antibody). C, Schematic showing the “Sholl” analysis of the outgrowth pattern. Circled samplings intersect with the neurite arbors providing information on how far the outgrowth can reach (represented as ending radius) and the number of branches (represented as number of intersections) at different distance from the soma (~30, 40‐105, and 120‐150 μm). D, Number and distribution of intersections at different distances from the soma. Note that the curve representing the mean number of intersections at 2% oxygen (blue line) is always above that at 20% oxygen (red line). E, Statistics showed that the intersection radius at 2% oxygen was significantly longer than at 20% oxygen. F, The sum of intersections of the whole cell was also significantly larger for 2% than for 20% oxygen. G, The initial branch number showed no significant difference between 2% and 20% oxygen. H, The magnitude of the difference in intersection number between 2% and 20% oxygen increased with distance from the soma. I Ramification index, which is the maximum number of intersections per sampling divided by the initial branch number, was significantly higher at 2% than at 20% oxygen (for box plot, *P < .05, **P < .01, and ***P < .001 by Mann‐Whitney test, n = 25 and 27 cells for 2% and 20% oxygen, respectively)

From the images of the primary neuron culture, the outgrowth appeared longer and denser at 2% oxygen (Figure 7A,B). The “Sholl” analysis showed that the ending radius (which represents the radius of neuronal outgrowth) was 44.4% longer at 2% than at 20% oxygen (*P* = .049; Figure 7E). This finding was consistent with the results obtained for the explant cultures (Figure 5).

The density of collateral branches was quantified by the number of intersections between the neurite arbor and concentric rings with regular radial increments centered in the neuronal soma (Figure 7C). The total number of intersections for each neuron was 2.13 times higher at 2% oxygen compared to 20% oxygen (*P* = .019) (Figure 7F). From the graph of the intersection distribution, it was evident that 2% oxygen always had a higher intersection number than 20% oxygen at various distances from soma, and this difference was even stronger at further distances from the soma (Figure 7D). Thus, the intersections were separately evaluated at distances from the soma of 0, ~30, 40‐105, and 120‐150 μm. Initial branch number (intersection number at 0 μm from soma) was 25% lower under 2% oxygen (*P* = .125) (Figure 7G), but at ~30 μm from the soma, the outgrowth density at 2% oxygen was already 20% higher than at 20% oxygen (*P* = .084). The density of outgrowth was 20% higher at distances from the soma of 40 to 105 μm (*P* = .001) and became 140% higher at 120 to 150 μm from the soma for 2% oxygen (*P* < .0001). Hence, in regions more distant from the soma, more significant increases of outgrowth density were observed under 2% oxygen (Figure 7H). The capability of collateral sprouting was represented by the ramification index parameter, which was 40% higher for 2% oxygen compared to 20% oxygen (*P* = .008; Figure 7I).

## 4 DISCUSSION

Nerve ingrowth inside painful IVD tissue has long been observed,^21^ but the underlying mechanism remains largely unknown. IVD degeneration is associated with nutrient deprivation and hypoxia stress in both IVD^17^ and DRG^24^ tissues. The hypothesis of this study was that the hypoxic stress in IVD and/or DRG causes aberrant neuronal outgrowth. In this study, the DRG‐derived cell line ND7/23 was used to screen the effect of hypoxia. DRG cells were exposed to hypoxia either directly or indirectly, by application of CM collected from IVDs cultured under hypoxia. Although no influence of hypoxia in IVD was found on DRG neurite outgrowth, hypoxia in DRG clearly evidenced longer neurites which were then validated using primary DRG cell and DRG explant culture. Since the length of regenerated nerve fibers largely determines the depth of the innervation, our results suggest that hypoxia in DRG may contribute to the aberrant neurite sprouting.

Another aspect of neuronal outgrowth is collateral sprouting which may lead to a higher innervation density. For primary DRG neuron cultures, in‐depth information could be obtained from the evaluation of neurite outgrowth pattern based on the “Sholl” method. Using this method for the examination of dendrite patterns in visual cortical slices, Talene et al observed that dendrites are confined within ~30 μm from the soma after full‐length Trk receptor transfection for pyramidal neurons, while truncated isoform T1 produced increase of dendritic mass at 40 to 105 μm from soma.^44^ Given the different aspects involved in sprouting, we also performed a pattern evaluation of neurite outgrowth at different distances from the soma. We found that density of outgrowth at 0 μm and within ~30 μm did not show any significant difference between 2% and 20% oxygen conditions, but the density of branches at 2% oxygen was significantly higher than at 20% oxygen in the region of 40‐105 μm to 120‐150 μm from the soma. Thus, the distal collateral sprouting under hypoxic conditions could contribute to the increased density of IVD innervation.

Despite the hypoxia‐promoted collateral sprouting observed at single cell level, the explant culture showed a 90.9% decrease of outgrowth frequency under hypoxia. It should be noted that the outgrowth frequency of the tissue explant depends not only on collateral sprouting of each single neuron, but also on the number of neurons with outgrowths. Neuronal necrosis in the hypoxic primary DRG neuronal cultures may have reduced the number of neurons with outgrowths, since early loss of plasma membrane integrity and staining by ethidium homodimer‐1 are key features of necrotic cell death.^45^

Hence, the changes in IVD innervation observed in painful clinical IVD tissue could potentially be explained by the balance of necrosis‐driven collapse of neuronal outgrowth and regenerative sprouting. Changes in the tissue innervation have been observed in other painful conditions. Poorly innervated (IVD and tendon) or neo‐formed (bone callus)^46^, ^47^ tissues with no initial nerve components can develop neoinnervation under stress. Meanwhile, in normally well‐innervated tissues like skin, nerve lesions may override the sprouting of nerve fibers and lead to reduced tissue innervation.^48^ Hence, it appears that stressful conditions can lead to either an increase or a decrease in tissue innervation. One evidence supporting the two‐sided effect of stress on nerve density in tissues comes from the study of complex regional pain syndrome, where both neo‐innervation (in hair follicles) and loss of innervation (in epidermal, sweat gland, and vessels) were simultaneously detected in skin biopsies.^49^

The axonal density of regenerative sprouting has been shown to be associated with neuropathic pain.^50^, ^51^ Denervation using surgical, physical, or chemical methods is frequently used to treat chronic LBP.^52^, ^53^ The increased density of nerve fibers in the tissue may sensitize the nociceptors to generate nociceptive pain. Neuropathic pain with less innervation, on the other hand, may arise through other mechanisms. For example, damaged neurons exhibit transcriptional changes that not only generate spontaneous action potentials,^54^ but also switch the phenotype of the DRG neurons. For instance, it has been shown that large DRG neurons begin to express calcitonin gene‐related peptide and tyrosine receptor kinase A (TrkA) when exposed to inflammatory signals and nerve growth factor (NGF).^55^

While direct exposure of DRGs to hypoxia influenced neurite outgrowth, no difference in neurite outgrowth was observed in DRGs cultured in hypoxia‐treated IVD CM and normoxia‐treated IVD CM. Several reasons can possibly explain the difference in neurite outgrowth response to direct and indirect exposure to hypoxia. First, when hypoxia is applied to an IVD, the DRG does not respond directly to hypoxia but to the factors released by the IVD as a result of hypoxia. Hence, the hypoxic stress is mediated by the IVD, thereby possibly decreasing its effect on DRG cells. Second, neural outgrowth is not the only parameter that can be affected by stressful stimuli in neurons, and other parameters (such as viability, neural sensitization, release of neurotransmitters, …) can be affected without noticeable changes in neural outgrowth. It is also possible that hypoxia alone indirectly via IVD is not enough to induce neurite sprouting. Third, our model has several limitations compared to the in vivo situation, such as the use of healthy IVDs, short culture time, and absence of mechanical loading (a key parameter for metabolic exchange). In fact, other mechanisms of IVD degeneration may play an essential role in promoting IVD mediated neurite ingrowth.^56^, ^57^ Finally, in the degenerative IVD, local oxygen concentration and oxygen gradients in the proximity of neurite endings may have a stronger influence than the response of whole IVDs to hypoxia as assessed in this in vitro model.

Neuronal necrosis represents injury. Necrotic cells, losing plasma membrane integrity, will release their intracellular content, thereby provoking inflammation.^58^ On the contrary, apoptosis is one kind of programmed cell death that has evolved to rapidly and efficiently eliminate unwanted cells, without eliciting inflammation and immune responses.^45^ Based on our results, the ND7/23 cell line viability was reduced because of a higher proportion of necrotic cells but not due to apoptosis, since the expression of anti‐apoptosis genes was even upregulated by hypoxia treatment. Similarly, a higher necrosis/apoptosis ratio was observed in primary DRG neuron cultures under hypoxic culture conditions. In vivo, the necrosis/apoptosis ratio has not been well characterized in DRGs yet, but the switch between necrosis and apoptosis represents an important tissue protecting mechanism against stress^59^ and has been widely investigated in other tissues. For example, in vivo brain ischemia models showed that the neural protective agent Prothymosin α leads to a necrosis‐apoptosis switch^60^ and in vivo pancreatitis models showed a correlation between the injury effect of hypoxia/oxidative stress and an increased necrosis/apoptosis transition.^61^ The characterization of the mechanism of neuronal death, whether apoptosis or necrosis, will greatly improve our understanding of neuropathic components in chronic LBP.^62^ According to International Association for the Study of Pain, neuropathic pain is defined as “pain that arises from a lesion or disease affecting the somatosensory system.”^63^ Indeed, neuronal apoptosis has been observed in in vivo neuropathic pain models,^64^ but since neuronal apoptosis can be a consequence of nearby cell necrosis,^65^ it remains unknown whether necrosis or apoptosis plays the main role in neuronal death related to neuropathic pain. Furthermore, the role of neuronal apoptosis in promoting pain remains uncertain, since mice deficient of pro‐apoptosis gene *Bax* showed even higher nociceptive and spontaneous pain behavior.^64^, ^66^ Although the in vitro model used in our study offers the advantage of studying the sole effect of hypoxia (excluding other factors), in vivo characterization of DRG neuronal death (necrosis/apoptosis ratio) in IVD degeneration requires further investigations.

Screening using a cell line can be an easy and useful tool for preliminary evaluation. The ND7/23 is a hybrid line produced by PEG‐mediated cell fusion of mouse neuroblastoma (N18tg2) and rat DRG neurons that has been used to study nociception and neuronal outgrowth.^30^, ^31^ In the present study, the same trends of neuronal outgrowth and viability were found in the cell line and primary neurons. Although the cell line was immortalized by a fusion with neuroblastoma, it may still function as a useful tool for preliminary screening provided proper validation using primary DRG and tissue explant culture.

Multiple mechanisms may have been involved in IVD neo‐innervation and chronic pain, such as inflammation^56^ and matrix degradation^67^ in the affected tissue. In fact, these factors are closely linked to each other. For example, hypoxia is a consequence and a distinctive feature of inflammation and can itself induce inflammation.^68^ These findings highlight the hypoxic microenvironment as a potential target in discogenic pain treatment among the multiple mechanisms involved.

## 5 CONCLUSIONS

In summary, neuronal outgrowth could be influenced by both neuronal necrosis and regenerative sprouting. At the tissue level, hypoxia increased the length but reduced the frequency of neuronal outgrowth; at the single viable neuron level, hypoxia promoted neuronal outgrowth length and collateral sprouting. The reduced outgrowth frequency at tissue level in hypoxia may arise from a higher neuronal necrosis. The balance between neuronal necrosis and regenerative plasticity may explain the contradictory increase or decrease of innervation in different clinical cases of chronic pain. Although hypoxia may play an important role in chronic pain, multifactorial mechanisms need further investigations.

## DISCLOSURE STATEMENT

The authors declare no potential conflict of interest.

## AUTHORS' CONTRIBUTION

Junxuan Ma, Mauro Alini, and Marianna Peroglio contributed to research design. Junxuan Ma, Despina Stefanoska, and Valentina Basoli acquired and analyzed the data. Laura S. Stone, Maria Hildebrand, Corrinus C. van Donkelaar, Xuenong Zou, and Sibylle Grad contributed to interpretation of data. Junxuan Ma, Despina Stefanoska, and Marianna Peroglio drafted the paper. All authors revised the paper critically and approved the submitted and final version.
